# Structure-Activity Relationship Studies of 4-((4-(2-fluorophenyl)piperazin-1-yl)methyl)-6-imino-N-(naphthalen-2-yl)-1,3,5-triazin-2-amine (FPMINT) Analogues as Inhibitors of Human Equilibrative Nucleoside Transporters

**DOI:** 10.3389/fphar.2022.837555

**Published:** 2022-02-21

**Authors:** Renkai Li, Winston Wing-Shum Mak, Jingjing Li, Chengwen Zheng, Polly Ho-Ting Shiu, Sai-Wang Seto, Simon Ming-Yuen Lee, George Pak-Heng Leung

**Affiliations:** ^1^ Department of Pharmacology and Pharmacy, University of Hong Kong, Pokfulam, Hong Kong SAR, China; ^2^ Department of Applied Biology and Chemical Technology, The Hong Kong Polytechnic University, Kowloon, Hong Kong SAR, China; ^3^ State Key Laboratory of Quality Research in Chinese Medicine, Institute of Chinese Medical Sciences, University of Macau, Avenida da Universidade, Taipa, China

**Keywords:** equilibrative nucleoside transporter, inhibitor, structure-activity relationship, FPMINT, mechanism of action

## Abstract

Equilibrative nucleoside transporters (ENTs) play a vital role in nucleotide synthesis, regulation of adenosine function and chemotherapy. Current inhibitors of ENTs are mostly ENT1-selective. Our previous study has demonstrated that 4-((4-(2-fluorophenyl)piperazin-1-yl)methyl)-6-imino-N-(naphthalen-2-yl)-1,3,5-triazin-2-amine (FPMINT) is a novel inhibitor of ENTs, which is more selective to ENT2 than to ENT1. The present study aimed to screen a series of FPMINT analogues and study their structure-activity relationship. Nucleoside transporter-deficient cells transfected with cloned human ENT1 and ENT2 were used as *in vitro* models. The results of the [^3^H]uridine uptake study showed that the replacement of the naphthalene moiety with the benzene moiety could abolish the inhibitory effects on ENT1 and ENT2. The addition of chloride to the meta position of this benzene moiety could restore only the inhibitory effect on ENT1 but had no effect on ENT2. However, the addition of the methyl group to the meta position or the ethyl or oxymethyl group to the para position of this benzene moiety could regain the inhibitory activity on both ENT1 and ENT2. The presence of a halogen substitute, regardless of the position, in the fluorophenyl moiety next to the piperazine ring was essential for the inhibitory effects on ENT1 and ENT2. Among the analogues tested, compound **3c** was the most potent inhibitor. Compound **3c** reduced *V*_max_ of [^3^H]uridine uptake in ENT1 and ENT2 without affecting *K*_m_. The inhibitory effect of compound **3c** could not be washed out. Compound **3c** did not affect cell viability, protein expression and internalization of ENT1 and ENT2. Therefore, similar to FPMINT, compound **3c** was an irreversible and non-competitive inhibitor. Molecular docking analysis also showed that the binding site of compound **3c** in ENT1 may be different from that of other conventional inhibitors. It is expected that structural modification may further improve its potency and selectivity and lead to the development of useful pharmacological agents.

## Introduction

Nucleoside transporters are transmembrane proteins responsible for transporting physiological nucleosides essential for the salvage pathways for the biosynthesis of nucleotides. They also participate in modulating the extracellular and intracellular concentrations of physiological nucleosides such as adenosine (Molina-Arcas et al., 2009), which participates in numerous essential physiological functions, including anti-inflammatory, cardioprotective and vasodilatory effects (Reiss et al., 2019). Moreover, they play an indispensable role in chemotherapy as they control the intracellular accumulation of anticancer and antiviral nucleoside analogues (Leung and Tse, 2007; Cano-Soldado and Pastor-Anglada, 2012). A major class of nucleoside transporters is equilibrative nucleoside transporters (ENTs), which are sodium-independent and can transport nucleosides along the concentration gradient (Baldwin et al., 2004). In humans, ENTs can be divided into four subtypes: ENT1–4. ENTs comprise hydrophobic α-helices across the cell membrane with an intracellular N-terminus and an extracellular C-terminus (Baldwin et al., 2004). ENT1 occurs in all tissues, such as the heart, brain, and lungs, with varying expression levels, whereas ENT2 is highly expressed particularly in skeletal muscles and adrenal gland (Griffiths et al., 1997; Crawford et al., 1998). Both ENT1 and ENT2 can transport purine and pyrimidine nucleosides. The affinities of ENT1 to thymidine, adenosine, cytidine and guanosine are 2.6-, 2.8-, 7.7- and 19.3-fold higher than those of ENT2, respectively (Ward et al., 2000). ENT3, like ENT1 and ENT2, is found in various tissues, but it is localised in intracellular organelles such as endosomes, lysosomes and mitochondria rather than in the cell membrane (Baldwin et al., 2005; Govindarajan et al., 2009; Liu et al., 2015). ENT3 also transports a wide range of nucleobases and nucleosides. Interestingly, its activity is optimal at an acidic pH (Baldwin et al., 2005). ENT4 is mainly expressed in the brain, heart and skeletal muscles (Barnes et al., 2006). It transports only adenosine, and its activity is increased in acidic environment. In addition to the location and substrate affinity, ENTs exhibit different pharmacological properties. ENT1 and ENT2 can be inhibited by conventional ENT inhibitors such as S-(4-nitrobenzyl)-6-thioinosine (NBMPR) and dipyridamole, but the IC_50_ values of ENT2 against these inhibitors are 7,000- and 71-fold higher than those of ENT1, respectively (Ward et al., 2000). In contrast, ENT3 and ENT4 are inert to NBMPR and dipyridamole (Baldwin et al., 2005; Barnes et al., 2006). ENT1 and ENT2 can also be inhibited by other compounds, including benzodiazepines, cilostazol, KF24345, propentofylline and troglitazone in the nanomolar to micromolar range (Patel et al., 1982; Parkinson et al., 1993; Liu et al., 2000; Seubert et al., 2000; Hammond and Archer, 2004; Leung et al., 2005). These compounds are also more selective to ENT1 than to other ENTs.

**FIGURE 1:**
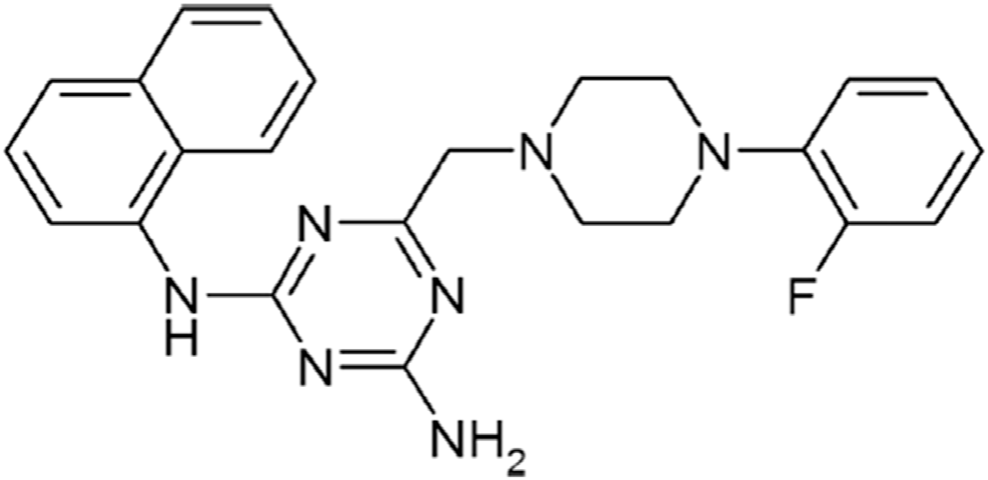
Chemical structure of FPMINT.

There has been a slow progress in the development of ENT2-selective inhibitors. Certain derivatives of draflazine are reported to be 2- to 6-fold more selective to ENT2 than to ENT1 (Hammond, 2000). Our previous study also showed that 4-((4-(2-fluorophenyl)piperazin-1-yl)methyl)-6-imino-N-(naphthalen-2-yl)-1,3,5-triazin-2-amine (FPMINT) (Figure 1) is 5- to 10-fold more selective to ENT2 than to ENT1 (Tang et al., 2016). The present study aimed to compare the potency and selectivity of different analogues of FPMINT to ENT1 and ENT2, and determine their structure-activity relationship. A representative analogue was selected for investigating its mechanism of action.

## Materials and Methods

### Materials

Culture media and supplements were purchased from Thermo Fisher Scientific (Waltham, MA). Antibodies were obtained from Alomone Labs (Jerusalem, Israel). Sulfo-NHS-SS-biotin was purchased from Thermo Fisher Scientific (Waltham, MA) [^3^H]uridine was purchased from PerkinElmer (Norwalk, CT, United States). FPMINT and its analogues were purchased from TopScience (Shanghai, China). Other chemicals were obtained from Sigma-Aldrich (St. Louis, MO, United States).

### PK15NTD/ENT1 and PK15NTD/ENT2 Cells

Nucleoside transporter-deficient porcine kidney fibroblast cells (PK15NTD) stably transfected with cloned human ENT1 or ENT2 (Ward et al., 2000) were used as the *in vitro* models. The cells were cultured in Dulbecco’s Modified Eagle’s Medium (DMEM) supplemented with 10% (v/v) foetal bovine serum, 0.5 mg/ml geneticin, 100 units/ml penicillin, 100 μg/ml streptomycin and 0.25 μg/ml of amphotericin B at 37°C in 5% CO_2_/95% air.

### Nucleoside Uptake Study

Confluent monolayers of PK15NTD/ENT1 and PK15NTD/ENT2 cells in 24-well plates were washed thrice with HEPES-buffered Ringer’s solution containing (mM): 135 NaCl, 10 glucose, 5 KCl, 5 HEPES, 3.33 NaH_2_PO_4_, 1.0 CaCl_2_, 1.0 MgCl_2_, 0.83 Na_2_HPO_4_; pH 7.4. The cells were incubated with different concentrations of FPMINT analogues (from 10 nM to 100 μM) containing [^3^H]uridine (2 μCi/ml, 1 μM) for 1 min. Passive uridine uptake was determined by incubating the cells in HEPES-buffered Ringer’s solution containing [^3^H]uridine in the presence of 0.5 mM NBMPR. The cells were rapidly washed five times with ice-cold phosphate-buffered saline (PBS). After air-drying, the cells were solubilised by overnight incubation in 500 μl of 5% Triton-X100. Next, 300 μl of the cell lysates were mixed with 2 ml of scintillation liquid. The radioactivity of each sample was quantified by a scintillation counter. The protein content in each well was determined spectrophotometrically using a commercial bicinchoninic acid assay (Pierce Biochemicals, Rockford, IL). Uptake values were expressed as picomoles of [^3^H]uridine per milligram of cellular protein per minute.

### Measurement of Cell Membrane Integrity and Cell Viability

PK15NTD/ENT1 and PK15NTD/ENT2 cells were seeded onto 96-well plates until they reached 80–90% confluent. The cells were then treated with different concentrations of compound **3c** or an equivalent volume of a vehicle (0.5% dimethyl sulfoxide (DMSO), as a control) in a serum-free medium for another 24 or 48 h. For measuring cell membrane integrity, the lactate dehydrogenase (LDH) released into the culture medium was measured with a detection kit (Abcam, Cambridge, UK) according to the manufacturer’s instructions. The absorbance at 490 nm was measured with a microplate absorbance reader. For measuring cell viability, 0.5 mg/ml methylthiazolyldiphenyl-tetrazolium bromide (MTT) was added to each well. The cells were further incubated for 4 h at 37°C to allow the formation of MTT formazan crystals. Finally, 100 μl of DMSO was added to lyse the cells and solubilise the crystals. The absorbance was measured at 560 nm, with the absorbance at 655 nm serving as the background.

### Western Blotting

PK15NTD/ENT1 and PK15NTD/ENT2 cells were grown to confluence in 10-cm culture dishes and treated with 50 µM of compound **3c** or an equivalent volume of a vehicle (0.5% DMSO) for 24 h or 48 h. The cells were washed thrice with ice-cold PBS. Total protein was extracted by cell lysis in a lysis buffer (5 mM sodium monophosphate, pH 8) with a protease inhibitor cocktail (Sigma, St Louis, MO, United States; 1:1,000 [v/v]). The protein content was measured by bicinchoninic acid assay. Protein samples were separated by 12.5% sodium dodecyl sulfate–polyacrylamide gel electrophoresis (SDS-PAGE) and blotted onto polyvinylidene difluoride (PVDF) membranes. The membranes were blocked with 5% (w/v) non-fat dry milk in PBS for 1 h at room temperature and then incubated with rabbit polyclonal anti-ENT1 or anti-ENT2 primary antibodies (Alomone Labs, Jerusalem, Israel) (1:200 [v/v] dilution in a blocking solution) at 4°C overnight. After washing thrice with PBS containing 0.02% (v/v) Triton X-100, the membranes were then incubated with horseradish peroxidase-conjugated goat anti-rabbit secondary antibodies (1:3,000 [v/v] dilution in a blocking solution) at room temperature for 2 h. The membranes were then again washed, and the bound secondary antibodies were detected using an enhanced chemiluminescence kit (Bio-Rad, Hercules, CA, United States). The antibodies recognized ENT1 as a protein of 40 kDa, and ENT2 as proteins of 50 and 47 kDa (Ward et al., 2000). To semi-quantify the ENT1 and ENT2 protein levels, the same membranes were washed three times with a stripping buffer for 10 min at room temperature, and the protein expression level of β-actin was measured using monoclonal mouse anti-β-actin primary antibodies (1:3,000 [v/v] dilution in a blocking solution). The antibody recognized β-actin as a protein of 42 kDa, The optical densities of ENT1 and ENT2 were normalised to that of β-actin.

### Cell Surface Biotinylation and Internalization Assay

To label all surface proteins, after washing with ice-cold PBS, PK15NTD/ENT1 and PK15NTD/ENT2 cells were incubated with sulfo-NHS-SS-biotin (300 μg/ml) for 30 min at 4°C to biotinylate cell surface proteins. The unbound biotin was washed away by PBS containing 0.1% BSA at 4°C. The biotinylated cells were then incubated with the original growth media with or without 50 µM compound **3c** and returned to the 5% CO_2_ incubator at 37 °C for 24 h or 48 h for transporter internalization. Transporter trafficking was stopped by rapidly cooling the cells at 4°C. Biotinylated proteins remaining on the cell surface were stripped by glutathione (150 mM glutathione, 150 mM NaCl, pH 8.75), but internalized transporters were protected and still contained biotin. Subsequently, 50 mM iodoacetamide in PBS was used to neutralize glutathione. Cells were then immediately solubilized in extraction buffer (50 mM Tris, pH 7.4, 150 mM NaCl, 1 mM EDTA, 1% Triton X-100, 0.5% sodium deoxycholate, 30 mM NaF, 1 mM sodium orthovanadate and protease inhibitor cocktail). After centrifugation at 13,000 x g for 5 min, supernatants with equal amounts of total proteins were agitated with streptavidin-agarose beads at 4°C overnight. Finally, the biotinylated transporters in the pull-down complexes, which should represent internalized transporters, were eluted by Laemmli buffer and resolved by SDS-PAGE as described above. Antibody against ENT1 or ENT2 was used to detect internalized transporters in the pull-down complexes and total transporter expressions in cell extracts.

### Molecular Docking Analysis

The X-ray crystal structure of human ENT1 was obtained from the Protein Data Bank (http://www.rcsb.org/pdb) (PDB ID: 6OB7). Two-dimensional structures of compound **3c** and draflazine were downloaded from the PubChem database (http://pubchem.ncbi.nlm.nih.gov) (CID: 666,635 and 60849, respectively) and was transformed into three-dimensional structure using Chem3D 19.0 software. The structures of ENT1, compound **3c** and draflazine were pretreated and standardized with the Protein Preparation Wizard module of Schrödinger Maestro suite 2015 and the LigPreg module of Schrödinger LigPrep suite 2015, respectively. Autodock Vina software and Lamarckian Genetic Algorithm program were used to analyse the molecular interactions between ENT1 and drugs with default parameters. ENT2 was not studied because its crystal structure is not available.

### Statistical Analysis

All data were analysed by GraphPad Prism software (San Diego, CA, United States). Data are expressed as mean ± standard error (SE) of three experiments performed in triplicate. The IC_50_ values were determined from the dose-response curves fitted with a logistic function. For the kinetic study of [^3^H]uridine uptake, apparent *K*_*m*_ and *V*_max_ values were determined from the Michaelis-Menten equation, and *K*_*i*_ was determined from the *X*-intercept of linear regression. Analysis of variance and Student’s *t-*test were performed for multiple and paired variables, respectively. *p* values of <0.05 were considered to be statistically significant.

## Results

### Dose-Response Relationship of FPMINT Analogues for ENT1 and ENT2

**FIGURE 2:**
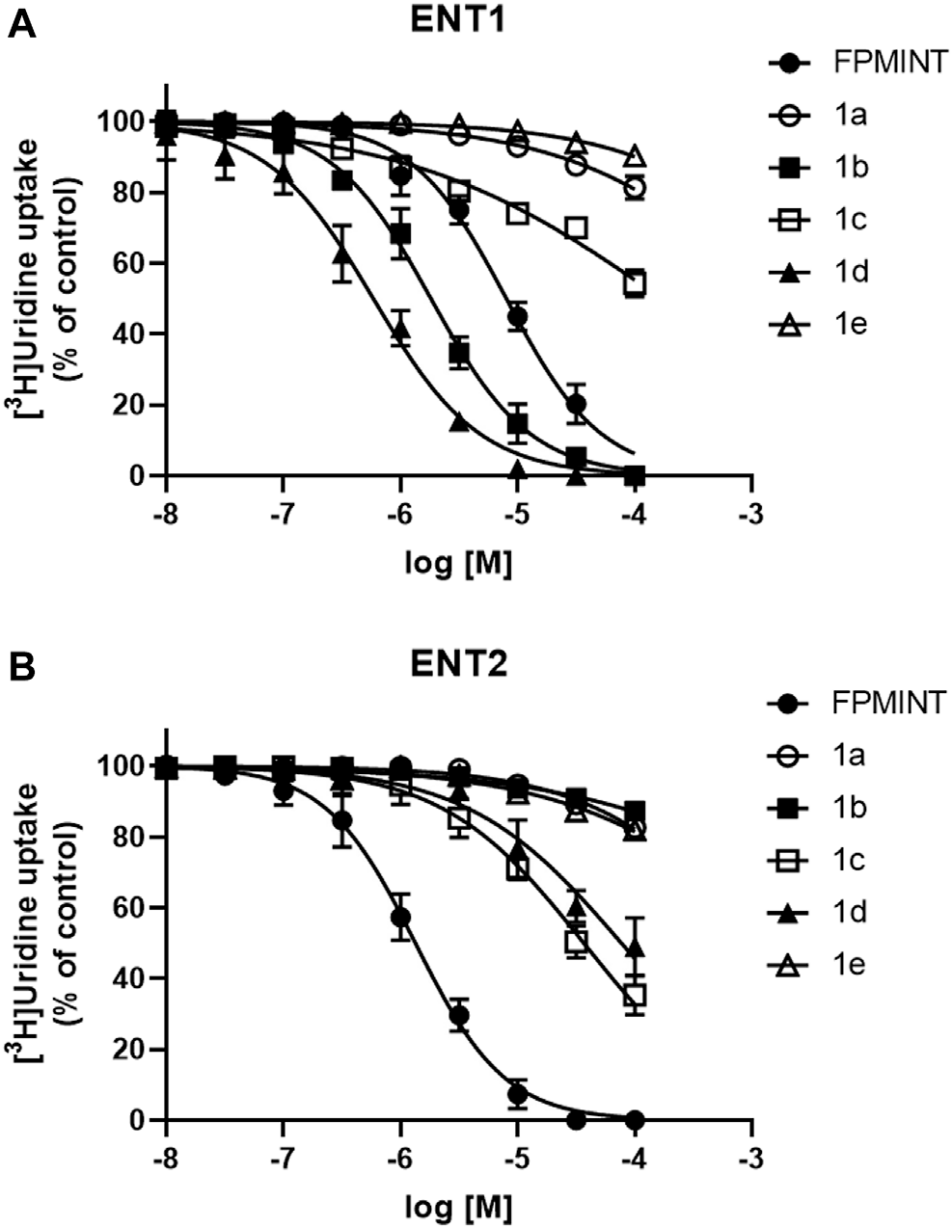
Effects of FPMINT analogues (listed in Supplementary Table S1) on [^3^H]uridine uptake by ENT1 and ENT2 [^3^H]uridine uptake (1 μM, 2 μCi/ml) in **(A)** PK15NTD/ENT1 and **(B)** PK15NTD/ENT2 cells was measured in the presence of various concentrations of FPMINT analogues listed in Supplementary Table S1 (10 nM–100 μM).

**FIGURE 3:**
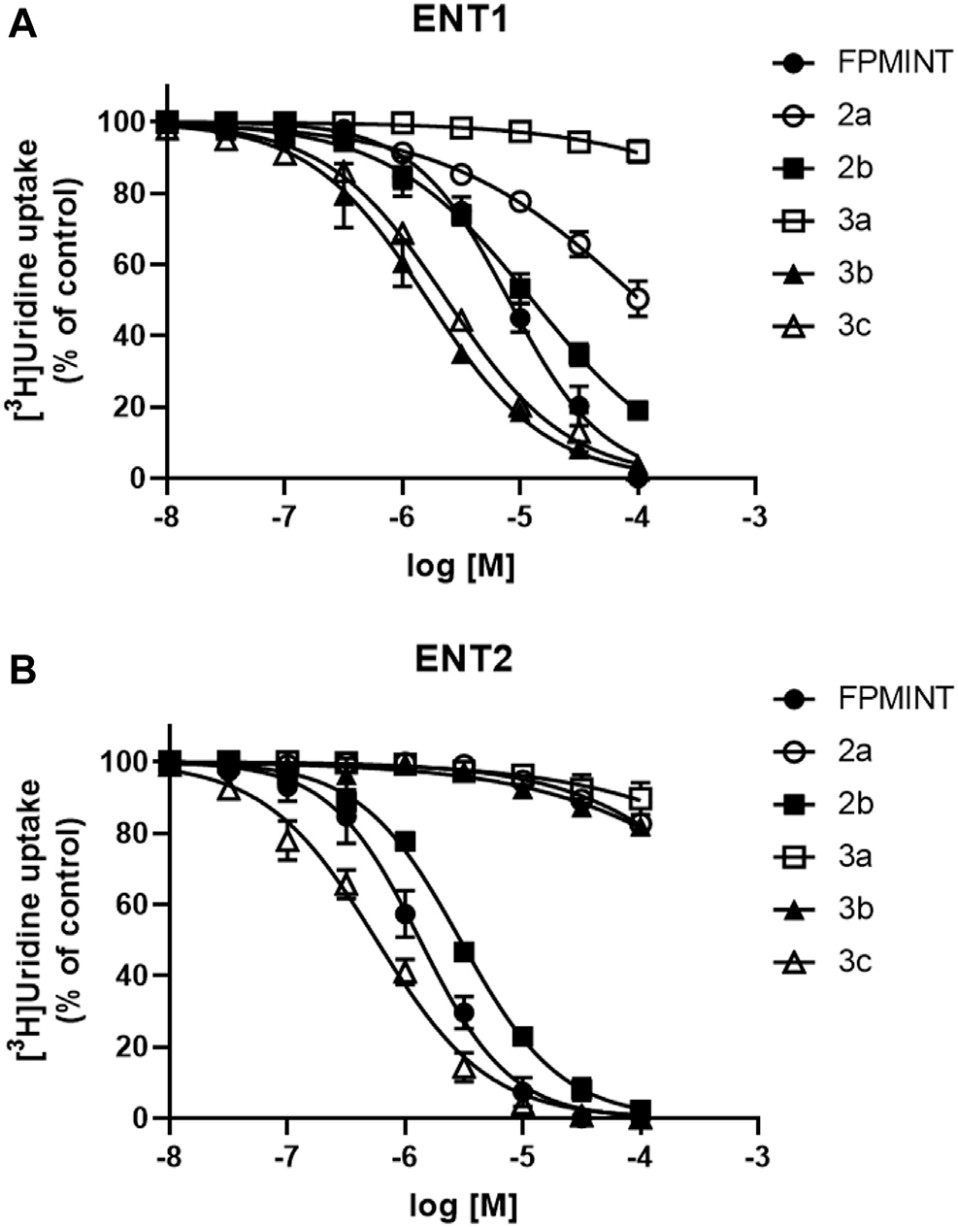
Effects of FPMINT analogues (listed in Supplementary Table S2) on [^3^H]uridine uptake by ENT1 and ENT2 [^3^H]uridine uptake (1 μM, 2 μCi/ml) in **(A)** PK15NTD/ENT1 and **(B)** PK15NTD/ENT2 cells was measured in the presence of various concentrations of FPMINT analogues listed in Supplementary Table S2 (10 nM–100 μM).

PK15NTD/ENT1 and PK15NTD/ENT2 cells were used to screen a series of FPMINT analogues listed in Supplementary Tables S1, S2. In control condition [^3^H]uridine uptake in PK15NTD/ENT1 and PK15NTD/ENT2 cells were 18.42 ± 2.93 pmol/mg protein/min and 61.35 ± 6.67 pmol/mg protein/min, respectively. The dose-response curves for each FPMINT analogue against ENT1 and ENT2 are shown in Figures 2, 3. Their IC_50_ values for ENT1 and ENT2 were shown in Supplementary Tables S1, S2.

Compounds **1a**, **1b, 1c**, **1d** and **1e** are the analogues of FPMINT, with modification in the N-naphthalene moiety. Compound **1a** and **1e** showed negligible effects on both ENT1 and ENT2. Compound **1b** inhibited ENT1 with an IC_50_ value of 1.82 µM, but it had no effect on ENT2. Similar to FPMINT, compound **1c** was more selective to ENT2 than to ENT1. The IC_50_ values of compound **1c** against ENT1 and ENT2 were 171.11 µM and 36.82 µM, respectively. Although the potencies of compound **1c** against ENT1 and ENT2 were lower than that of FPMINT, the selectivities of FPMINT and compound **1c** were comparable (5.99-fold and 4.647-fold more selective to ENT2 for FPMINT and compound **1c**, respectively). Unlike compound **1c**, compound **1d** was more selective to ENT1 than to ENT2 with IC_50_ values of 0.59 µM and 77.12 µM, respectively.

Compounds **2a**, **2b**, **3a**, **3b** and **3c** are the analogues of FPMINT, with changes in the N-naphthalene moiety and/or the fluorophenyl moiety. Compound **3a** had no effect on both ENT1 and ENT2. Compounds **2a** and **3b** inhibited ENT1 with IC_50_ values of 104.92 µM and 1.65 µM, respectively. However, they did not inhibit ENT2. Compounds **2b** and **3c** inhibited both ENT1 and ENT2, but they were relatively more selective to ENT2. The IC_50_ values of compounds **2b** and **3c** against ENT1 were 12.68 µM and 2.38 µM, respectively, whereas their IC_50_ values against ENT2 were 2.95 µM and 0.57 µM, respectively. Compared to FPMINT, compound **3c** showed a stronger potency, but compound **2b** showed a weaker potency. However, their selectivities were close to that of FPMINT (4.29-fold and 4.17-fold more selective to ENT2 for compounds **2b** and **3c**, respectively).

### 3.2 Effects of Compound 3c on the Transport Kinetics of ENT1 and ENT2

**FIGURE 4:**
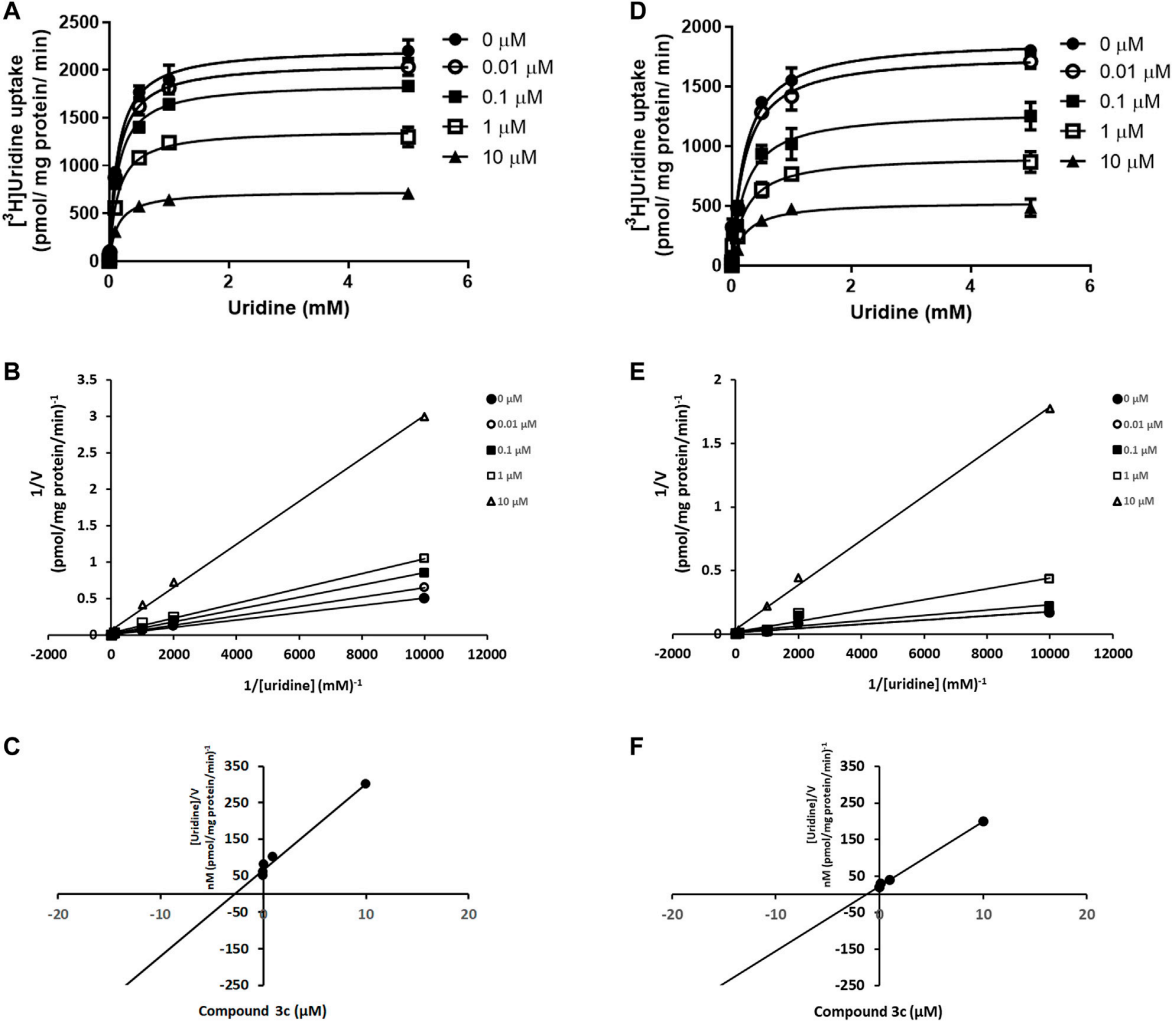
Competitive inhibition of ENT1 and ENT2 by compound 3c. Kinetic study of [^3^H]uridine uptake (0.1 μM–1 mM) was measured in **(A)** PK15NTD/ENT1 and **(D)** PK15NTD/ENT2 cells in the presence of various concentrations of compound **3c** (0, 0.01, 0.1, 1 and 10 µM) **(B,E)** 1/*V* versus 1/[S] plots of each curve in **(A)** and **(D)**, respectively **(C,F)** The plot of slopes of each line in **(B)** and **(E)**, respectively, versus the concentration of compound **3c**. Data are presented as mean ± SE of three experiments.

Compound **3c** was selected for further investigation. The kinetic study of [^3^H]uridine transport was performed by measuring the concentration-dependent uptake of [^3^H]uridine. This study could provide clue about the mode of action of compound **3c**. The uptake of [^3^H]uridine by ENT1 and ENT2 was saturable and conformed to Michaelis-Menten kinetics. Compound **3c** decreased the *V*_max_ of ENT1 but had no effect on *K*_*m*_. Specifically, the apparent *V*_max_ values were 2236 ± 56.32, 2083 ± 36.80, 1867 ± 27.63, 1377 ± 37.81 and 732.9 ± 12.02 (pmol/mg protein/min) and the *K*_*m*_ values were 0.143 ± 0.018, 0.142 ± 0.012, 0.143 ± 0.010, 0.144 ± 0.019 and 0.142 ± 0.011 (µM) in the presence of 0, 0.01, 0.1, 1 and 10 µM compound **3c**, respectively (Figure 4A). The slopes of the linear 1/*V* versus 1/[S] plots increased with the concentration of compound **3c** (Figure 4B). As observed from plotting the slopes against the concentration of compound **3c**, the *K*_*i*_ of compound **3c** for ENT1 (x - intercept = -*K*_*i*_) was 2.79 μM (Figure 4C). Similarly, compound **3c** decreased the *V*_max_ of ENT2 but did not affect *K*_*m*_. The apparent *V*_max_ values were 1895 ± 69.32, 1775 ± 76.83, 1295 ± 78.09, 918.6 ± 47.48 and 534.6 ± 28.21 (pmol/mg protein/min) and the *K*_*m*_ values were 0.220 ± 0.035, 0.220 ± 0.041, 0.228 ± 0.059, 0.229 ± 0.050 and 0.226 ± 0.051 (µM) in the presence of 0, 0.01, 0.1, 1 and 10 µM compound **3c**, respectively (Figure 4D). Similar to that for ENT1, the double reciprocal plot demonstrated that the slope was proportionally increased with the concentration of compound **3c** (Figure 4E), and the *K*_*i*_ value of compound **3c** for ENT2 was 0.54 µM (Figure 4F).

### Effects of Compound 3c on Cytotoxicity and Protein Expression Levels of ENT1 and ENT2

**FIGURE 5:**
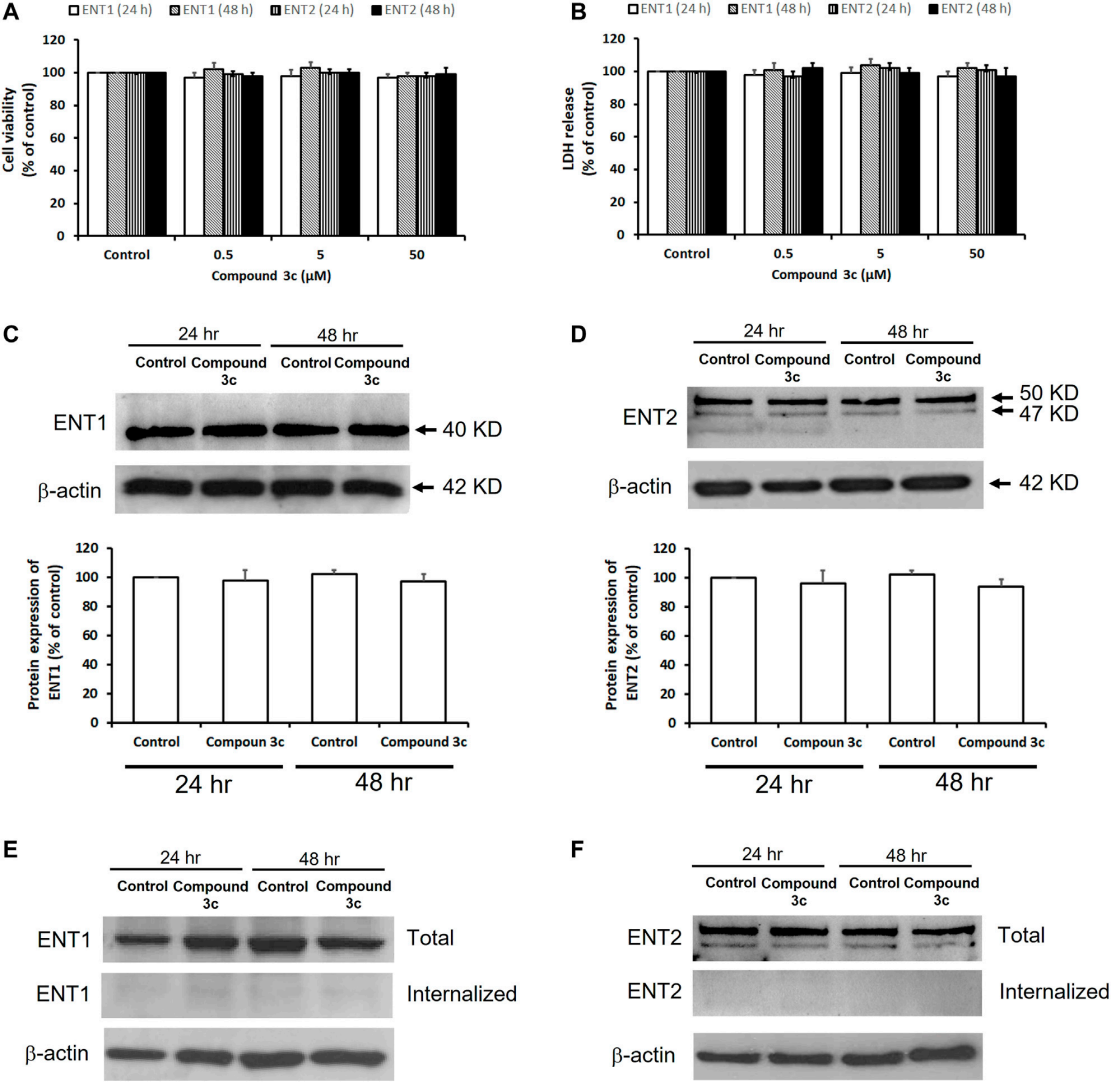
Effects of compound 3c on cytotoxicity and protein expressions of ENT1 and ENT2. Cytotoxicity of compound **3c** was measured after 24 h or 48 h treatment of PK15NTD/ENT1 and PK15NTD/ENT2 cells with various concentrations of compound **3c** (0.5, 5 and 50 μM) or a vehicle (0.5% DMSO, as a control) **(A)** The cell viability and **(B)** cell membrane integrity were determined by the MTT assay and LDH release, respectively **(C)** PK15NTD/ENT1 and **(D)** PK15NTD/ENT2 cells were incubated with 50 μM of compound **3c** or a vehicle (0.5% DMSO, as a control) for 24 or 48 h. Western blotting assay was performed to determine the protein expression levels of ENT1 and ENT2, with β-actin as an internal reference. Cell surface proteins of **(E)** PK15NTD/ENT1 and **(F)** PK15NTD/ENT2 cells were biotinylated and treated with 50 μM of compound **3c** or a vehicle (0.5% DMSO, as a control) for 24 or 48 h. After cleavage of extracellular biotin, internalized biotinylated proteins were precipitated with immobilized streptavidin beads and detected with an anti-ENT1 and anti-ENT2 antibodies by western blotting. Total ENT1 and ENT2 from whole-cell lysates were also detected for a comparison. β-actin served as an internal reference. Representative blots are from three independent experiments. Data are presented as mean ± SE of three experiments.

The effects of compound **3c** on cell viability and cell membrane integrity of PK15NTD/ENT1 and PK15NTD/ENT2 cells were studied by MTT assay and LDH release measurement, respectively. No significant changes in cell viability and cell membrane integrity were observed when PK15NTD/ENT1 or PK15NTD/ENT2 cells were incubated with 50 µM of compound **3c** for 24 or 48 h (Figures 5A,B). In western blotting assay, the protein expression levels of ENT1 and ENT2 were unaltered after treatment with 50 µM of compound **3c** for 24 h or 48 h (Figures 5C,D). Moreover, biotinylated ENT1 and ENT2 were exclusively found at the plasma membrane of PK15NTD cells upon the incubation with 50 µM compound **3c** for 24 h or 48 h (Figures 5E,F), suggesting that ENT1 and ENT2 did not undergo internationalization.

### Irreversible Inhibition of ENT1 and ENT2 by Compound 3c

**FIGURE 6:**
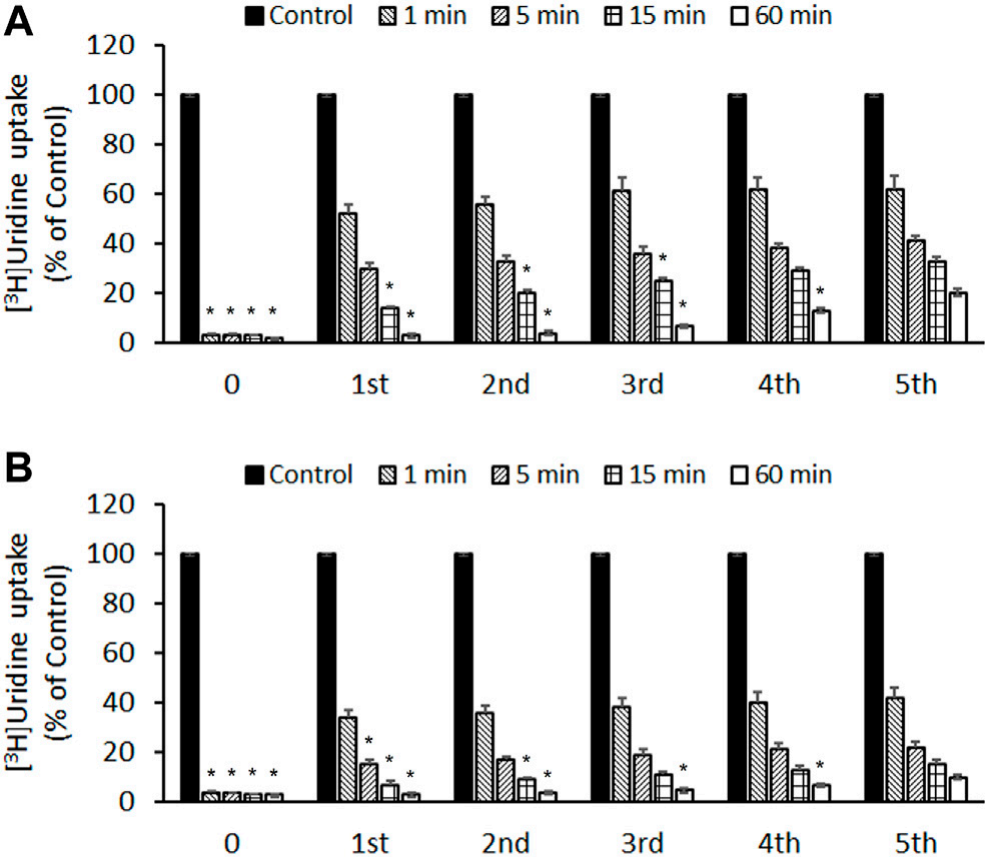
Reversibility of the inhibitory effects of compound 3c on ENT1 and ENT2 **(A)** PK15NTD/ENT1 and **(B)** PK15NTD/ENT2 cells were incubated with 50 μM of compound **3c** or a vehicle (0.5% DMSO, as a control) for different time (0–60 min) The cells were then washed five times. After wash-out for every single time [^3^H]uridine uptake (1 μM, 2 μCi/ml) was measured. Data are presented as mean ± SE of three experiments. ^*^ *p* < 0.05 compared with the fifth wash.

To determine whether the inhibitory effects of compound **3c** were reversible or irreversible, PK15NTD/ENT1 and PK15NTD/ENT2 cells were incubated with 50 µM of compound **3c** for different time periods (1–60 min). The cells were then washed five times with buffer. After wash-out for every single time [^3^H]uridine uptake was measured. The inhibitory effects of compound **3c** on both ENT1 and ENT2 were only partially reversed by washing (Figure 6). The longer the incubation time with compound **3c**, the more difficult it was to wash-out the inhibitory effects of compound **3c**.

### Molecular Interaction Between Compound 3c and ENT1

A molecular docking model was used to simulate the binding between compound **3c** and ENT1.

**FIGURE 7:**
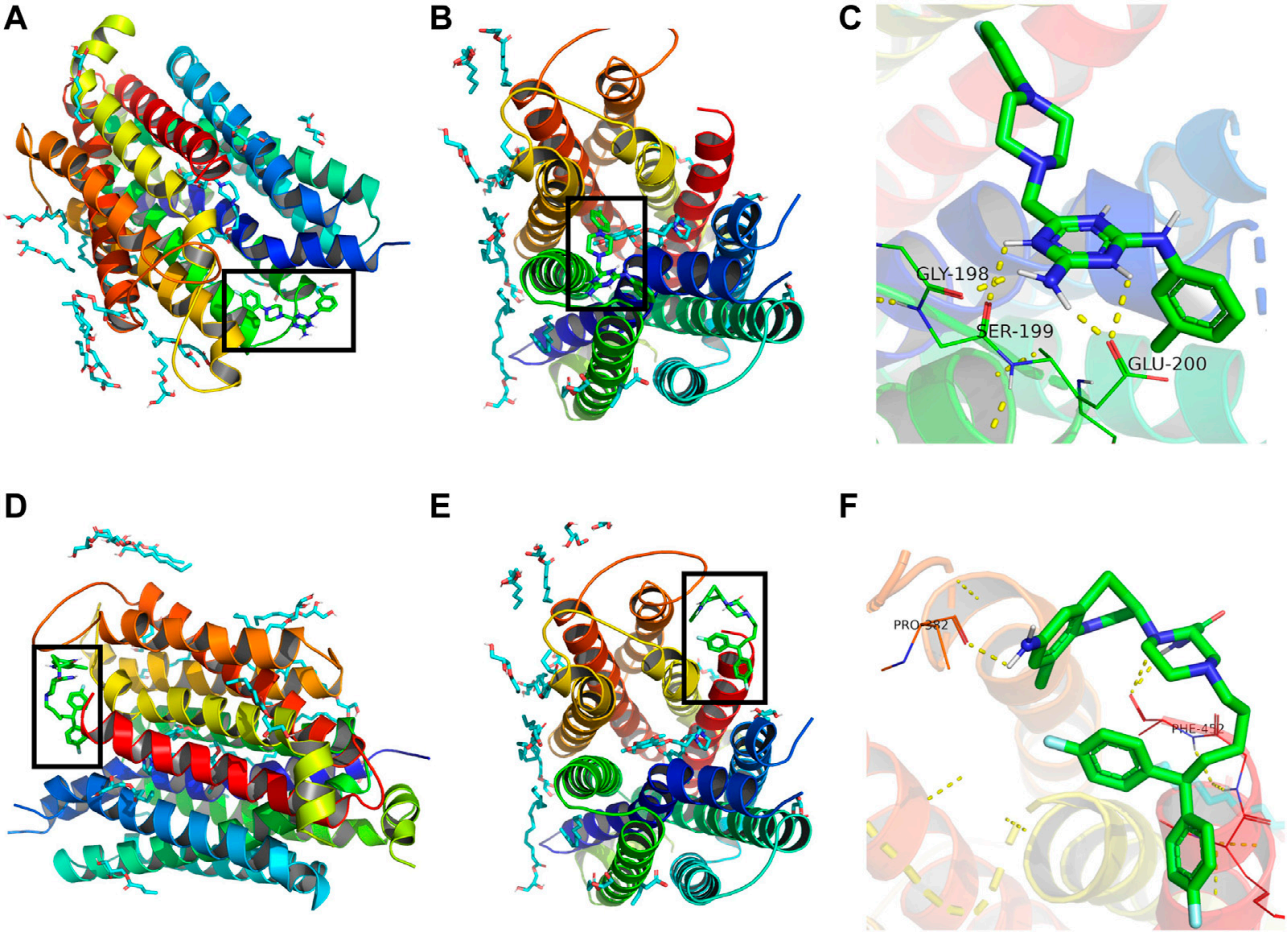
Molecular docking analysis of compound 3c against ENT1. 3D structural overview of ENT1 with **(A,B)** compound **3c** and **(D,E)** draflazine docked complexes. The black box showed the location of compound **3c.** A close-up of the docked protein displaying the amino acid residues of ENT1 involved in the binding with **(C)** compound **3c** and **(F)** draflazine.

Binding energy less than 0 kcal/mol indicates effective binding. The lower the binding energy, the more stable is the interaction between the drug molecule and protein. The binding energy of compound **3c** to ENT3 was -7.05 kcal/mol, suggesting that they could bind stably. The optimal interaction could be visualized by PyMoL software. Draflazine was used for comparison as it contains a piperazine ring as compound **3c**. The results showed that compound **3c** could form hydrogen bonds with Gly198, Ser199and Glu200 of ENT1 (Figures 7A–C) but draflazine from hydrogen bonds with Pro384 and Phe452 (Figures 7D–F). The piperazine rings in compound **3c** and draflazine did not form stable interaction with ENT1.

## Discussion

ENTs play an essential role in regulating the transport of physiological nucleosides and nucleoside analogues. The implications of pharmacological inhibitors of ENTs as therapeutic agents and research tools have been proposed for years. The most representative inhibitors of ENTs are NBMPR, dipyridamole and dilazep, which are 7,000-, 71- and 502-fold more potent, respectively, in inhibiting ENT1 than in inhibiting ENT2 (Ward et al., 2000; Playa et al., 2014). Some of their structural analogues were more potent against ENT1, but some were more potent against ENT2 than the parent compounds (Gupte and Buolamwini, 2004; Gupte and Buolamwini, 2007; Lin and Buolamwini, 2007; Playa et al., 2014). However, no ENT2-selective inhibitor could be identified from these analogues. Draflazine is another potent ENT1-selective inhibitor. Interestingly, a structure–activity study has demonstrated that several analogues of draflazine are 2- to 6-fold more selective towards ENT2 (Hammond, 2000). We have also previously reported that FPMINT is approximately 5- to 10-fold more selective to ENT2 than to ENT1 (Tang et al., 2016). In the present study, a series of FPMINT analogues with structural modification on the N-naphthalene moiety and the phenyl moiety were screened. We hope to establish a useful structure-activity relationship to guide the future design of more potent and ENT2-selective inhibitors.

First, the change from the naphthalene moiety to the benzene moiety abolished the effect on ENT1. This finding was confirmed based on the data of compounds **1a**, **1e** and **3a**. A possible reason was that the naphthalene moiety of FPMINT may provide a suitable conformation for fitting into the hydrophobic pocket of ENT1 and interact well with a specific binding site. However, the data of compounds **1b**, **1c**, **1d**, **3b** and **3c** suggested that the addition of a substitute to either the meta or para position of this benzene ring could restore the inhibitory activity on ENT1. These substitutes may facilitate the binding of compounds to ENT1. Interestingly, the analogue with an oxymethyl substitute at the para position of this benzene ring (i.e., compound **1d**) was much stronger than the analogue with an ethyl substitute (i.e., compound **1c**). Moreover, the analogue with a chloride substitute at the meta position of this benzene ring (i.e., compound **3b**) was slightly stronger than the analogue with a methyl substitute (i.e., compound **3c**). The reason for this phenomenon is not known, but a common characteristic of the chloride and oxymethyl substitutes is that they possess lone pairs of electrons, which may increase the interaction with certain functional groups in the binding site of ENT1 through hydrogen bonds.

Another determinant for the inhibitory effect on ENT1 is related to the fluorophenyl moiety next to the piperazine ring. The removal of the fluoride substitute from the fluorophenyl moiety (i.e., compound **2a**) reduced but not abolished the inhibitory effect on ENT1. Replacing the fluoride substitute at the ortho position with the chloride substitute at the meta position (i.e., compound **2b**) did not significantly affect the inhibitory effect on ENT1. Moreover, comparison of the results of compounds **1b** and **3b** showed that changing the fluoride substitute from the ortho position to the para position did not alter the inhibitory effect on ENT1. It is apparent that the presence of a halogen substitute, regardless of its position on the phenyl moiety, was critical to the inhibitory effect on ENT1, probably through the electrostatic interaction with the binding site.

The presence of a halogen substitute on the fluorophenyl moiety played an even more critical role in the inhibitory effect on ENT2 because the absence of fluoride (i.e., compound **2a**) not only decreased but also completely eliminated the inhibitory effect on ENT2. Similar to that for ENT1, the change of the fluoride substitute at the ortho position to the chloride substitute at the meta position (i.e., compound **2b**) restored the inhibitory effect on ENT2. Modification of the N-naphthalene moiety also greatly affected the inhibitory effect on ENT2. The replacement of the naphthalene moiety with the benzene moiety (i.e., compound **1a**) not only abolished the inhibitory effect on ENT1 but also on ENT2. In contrast to that for ENT1, the addition to a chloride substitute to the meta position of the benzene ring could not restore the inhibitory effect on ENT2. This assumption was clarified when we compared compounds **1a** against **1b**, and compounds **3a** against **3b**. However, the addition of the ethyl or oxymethyl group to the para position of this benzene ring (i.e., compounds **1c** and **1d**) enabled the compounds to regain the inhibitory activity, although the inhibitory effect was smaller than that of FPMINT. It remains unclear whether the presence of a para-substitute facilitates the binding of the compounds to ENT2. However, we believe that the methyl group in this benzene moiety may also be of paramount importance. The reason is that the change of the chloride substitute at the meta position of this benzene moiety (i.e., compound **3b**) to the methyl substitute (i.e., compound **3c**) could drastically make the compound, which was originally insensitive to ENT2, to become highly sensitive to ENT2.

It is of interest to note the important role of this methyl group in the pharmacological effect of compound **3c**. Addition of methyl group has been known to drastically increase the potency of many pharmacological agents (Quancard et al., 2012; O’Reilly et al., 2013). This so-called “magic methyl effect” in medicinal chemistry could be attributed to several physical phenomena. First, when a drug molecule binds to a hydrophobic protein cavity in an aqueous environment, the hydrophobic property of the methyl group reduces the desolvation energy required to remove the solvation by water molecules (Eugene Kellogg and Abraham, 2000; Southall et al., 2002). Second, a small increase in hydrophobicity may facilitate a nonpolar interaction in a certain part of the binding site (Boehringer et al., 2010). Moreover, the steric hindrance due to the methyl group can prevent the enzymatic degradation and hence prolong the half-life of drugs. However, such metabolic stability was not applicable to our *in vitro* model. Furthermore, the methyl group can increase the rotational barrier and lock the arene moiety in its axial position. The decreased conformational flexibility may cause burial of both the phenyl and methyl groups into the hydrophobic pockets within the active site, leading to increase in potency.

Compound **3c** was stronger than FPMINT in terms of the inhibitory potency, although both compounds were approximately 5-fold more selective to ENT2 than to ENT1. Among all the analogues tested, the chemical structure of compound **3c** was also the most different from that of FPMINT. This structural difference may lead to compound **3c** to have binding sites and hence the mechanism of action different from those of FPMINT. The parameters used in kinetic studies of nucleoside transport, such as *V*_max_, which represents the maximal transport rate, and *K*_*m*_, which represents the affinity for the transporters, may provide some hints to the mechanism of action of compound **3c**. Similar to FPMINT (Tang et al., 2016), compound **3c** decreased the *V*_max_ of ENT1-and ENT2-mediated uridine transport but did not change the apparent *K*_*m*_, reflecting that compound **3c** should be a non-competitive inhibitor. The inhibitory effect of compound **3c** could not be washed out, implying that the interaction between compound **3c** and ENTs was irreversible. Moreover, the inhibitory effect of compound **3c** occurred immediately without the need of pre-incubation. The western blot assay results also showed that compound **3c** did not downregulate the protein expression levels of ENT1 and ENT2, suggesting that compound **3c** directly acted on ENT1 and ENT2 and suppressed their transport activities. The biotinylation study also demonstrated that compound **3c** did not induced internalization of ENT1 or ENT2. It is therefore apparent that compound **3c** and FPMINT shared similar mechanism of action. Such irreversible and non-competitive mode of inhibition by compound **3c** and FPMINT were contrasting to that of other conventional ENT inhibitors such as NBMPR and dipyridamole, which inhibited ENTs competitively and reversibly (Jarvis, 1986; Kwong et al., 1987; Tang et al., 2016). Theoretically, the non-competitive and irreversible inhibition may be a potential advantage because the duration of action can be prolonged.

Draflzaine can inhibit the binding of NBMPR (Hammond., 2000), which occupies the central cavity of ENT1 (Wright and Lee, 2019). Kinetic studies have also suggested that draflazine and its analogues can compete with NBMPR for the same binding site in ENT1 (Vlachodimou et al., 2020). Same as draflazine, FPMINT and its analogues including compound **3c** contain a piperazine moiety. Therefore, it is not impossible that FPMINT and its analogues may share the same binding sites in ENT1 with draflazine, dialzep and NBMPR. Studies based on human and rat chimeras have revealed that transmembrane domains 3–6 play a significant role in hENT1 functionality in both substrate recognition and binding of inhibitors such as NBMPR, dipyridamole and dilazep (Sundaram et al., 1998; Sundaram et al., 2001). Moreover, the recent published crystal structure of ENT1 has indicated that the trimethoxybenzoic acid group of dilazep interacts with Trp29 and Gln158 in the orthosteric site of ENT1. The central diazepane ring of dilazep interacts with Met33 and the other trimethoxyphenyl ring interacts with Phe307 and Phe334 and Asn338. The ribose moiety of NBMPR interacts with Arg345 and Asp34, whereas the N-1 and N-3 amino groups of the thioinosine moiety of NBMPR interacts with Gln158 of ENT1. Furthermore, the purine moiety of NBMPR is surrounded by Leu26, Met89, Leu92 and Leu442 of ENT1 (Wright and Lee, 2019). Interestingly, our molecular docking results suggested that compound **3c** may form hydrogen bonds with Gly198, Ser199 and Glu200, which are supposed to be located at the junction of transmembrane domain 5 and the extracellular loop of ENT1 (Rehan et al., 2019), which is different from the proposed binding sites for NBMPR and dilazep. Moreover, the docking results suggested that draflazine formed stable interaction with Pro384 and Phe452, which is close to the binding sites for NBMPR and dilazep but far from that for compound 3c. The docking results also showed that, for both compound 3c and draflazine, the interaction between piperazine moiety and ENT1 was indeed not significant. Therefore, it is unlikely that draflazine and compound **3c** share similar binding sites although both contain a piperzaine moiety. Molecular docking could not be carried out for ENT2 since its crystal structure is not available. Nonetheless, based on the alignment of amino acid sequence of ENT1 and ENT2, the corresponding amino acid residues for Gly198, Ser199 and Glu200 of ENT1 are Gly185, Val186 and Asp187 of ENT2. These amino acid residues are partly conserved. Serine is polar but valine is non-polar. However, glutamic acid and aspartic acid are similar as they are acidic amino acids, which are negatively charged at neutral pH. It is not impossible that these amino acid residues may also be involved in the binding on compound **3c** to ENT2. Further studies are warranted to find out the exact binding sites for compound **3c** in ENT2.

It has been reported that certain piperazine derivatives may have cytotoxic effects on hepatocytes and cancer cell lines (Koksal Akkoc et al., 2012; Dias-da-Silva et al., 2015). Although the piperazine ring is present in the chemical structures of FPMINT and compound **3c**, they are not cytotoxic to PK15NTD cells. Nevertheless, further experiments are still required to completely reveal their safety profile. It is believed that the optimisation of the chemical structures of FPMINT and compound **3c** may further improve the selectivity to ENT2. Development of a selective ENT2 inhibitor will be a useful research tool or even a potential therapeutic agent. ENT2 is particularly abundant in skeletal muscle and adrenal gland (Griffiths et al., 1997; Crawford et al., 1998) but its functions in these tissues remain obscure. Compared to wild-type mice, ENT2-knockout mice have mitigated pulmonary oedema, improved gaseous exchange, and better survival rate during acute lung injury (Eckle et al., 2013). Moreover, the deletion of ENT2 can protect mice against neuroinflammation and blood-brain barrier dysfunction induced by lipopolysaccharides (Wu et al., 2020). These effects are likely due to the increased extracellular level of adenosine after the deletion of ENT2 as adenosine has anti-inflammatory function. Although ENT2-knockout mice are an important model for investigating the physiological roles of ENT2, we cannot exclude the possibility that other nucleoside transporters may be activated or up-regulated as a compensatory mechanism in the animal, which may interfere with data interpretation. If this is the case, the use of selective ENT2 inhibitors may serve as an alternative approach.

In conclusion, our study has provided evidence that the modification of the N-naphthalene moiety and the fluorophenyl moiety in FPMINT can change the inhibitory potency and selectivity for ENT1 and ENT2. The insights obtained from the structure-activity relationship may be useful in designing and developing more potent and selective ENT2 inhibitors.

## Data Availability

The original contributions presented in the study are included in the article/Supplementary Material, further inquiries can be directed to the corresponding author.

## Supplementary Material

Supplementary Table S1 ENT1 and 2 inhibitory activities of FPMINT analogues with modification in the N-naphthalene moiety.

Supplementary Table S2 ENT1 and 2 inhibitory activities of FPMINT analogues with modification in the N-naphthalene moiety and/or the fluorophenyl moiety.
