# Supplementary figures and images for: Structure-Activity Relationship Studies of 4-((4-(2-fluorophenyl)piperazin-1-yl)methyl)-6-imino-N-(naphthalen-2-yl)-1,3,5-triazin-2-amine (FPMINT) Analogues as Inhibitors of Human Equilibrative Nucleoside Transporters
Source: Front Pharmacol. 2022 Feb 21;13:837555. doi: 10.3389/fphar.2022.837555 (PMC8899516; doi:10.3389/fphar.2022.837555)

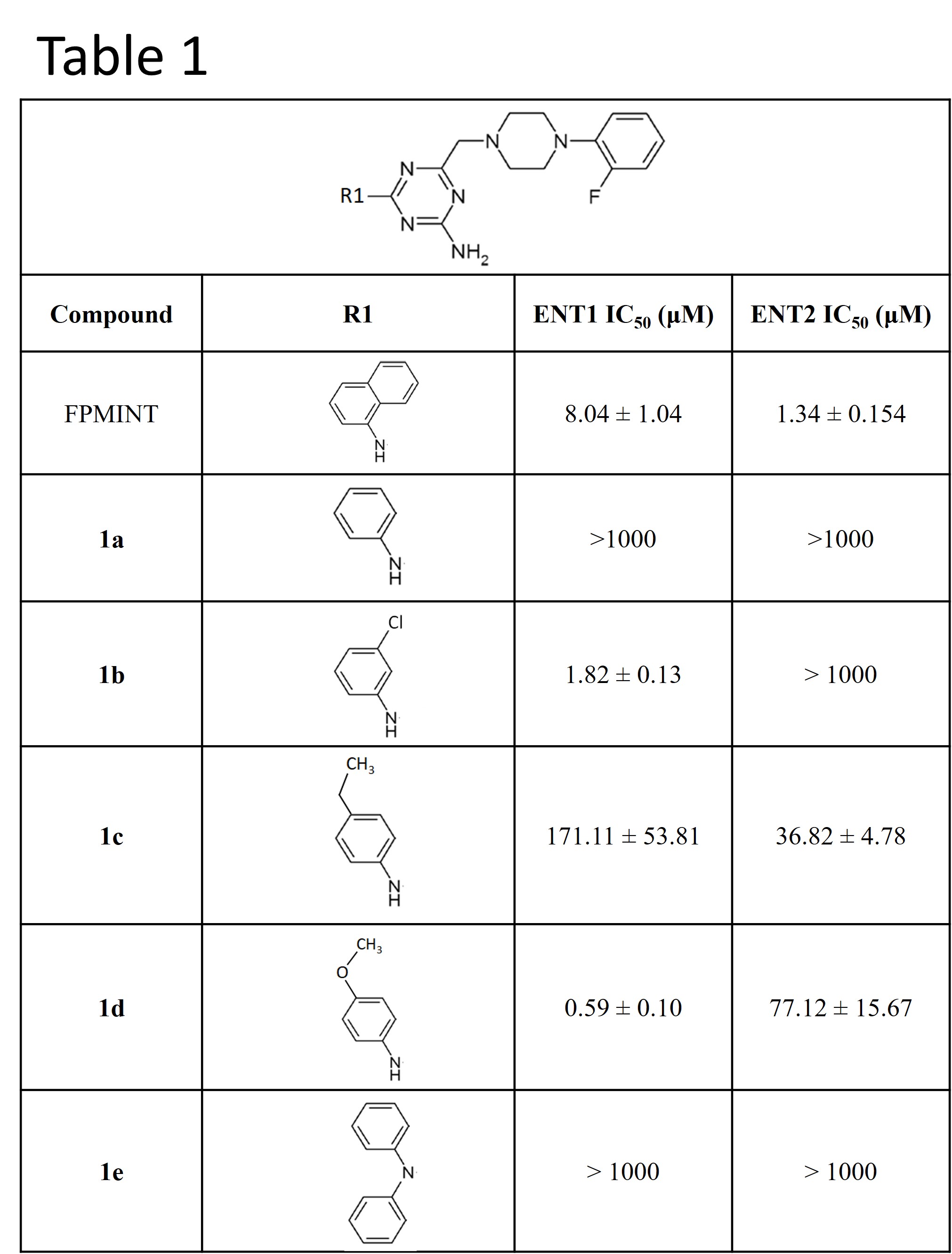

Supplement: Supplementary file 1 [file Image1.JPEG]

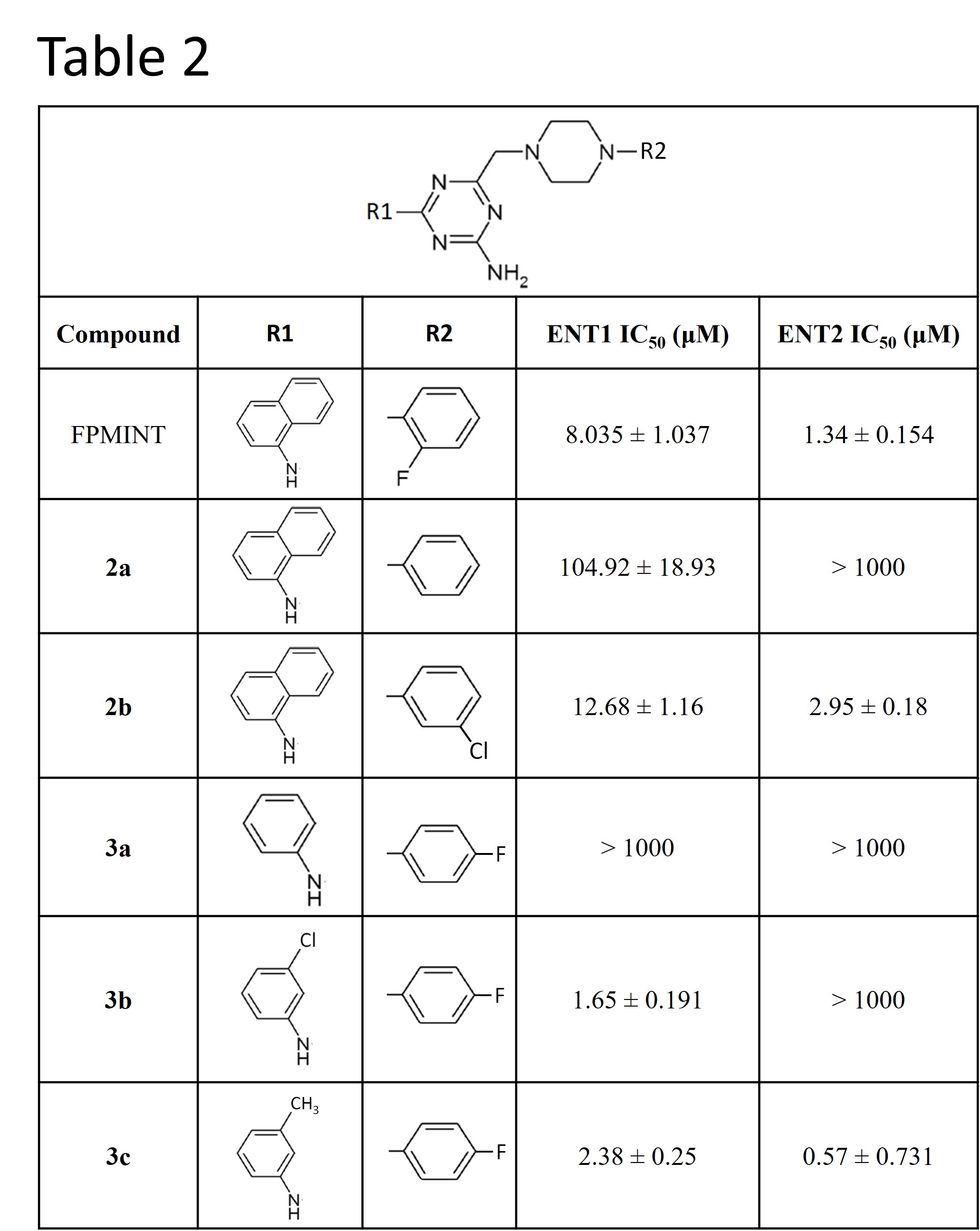

Supplement: Supplementary file 2 [file Image2.JPEG]
